# The crystallography of Pluto

**DOI:** 10.1107/S205225252001163X

**Published:** 2020-08-29

**Authors:** Christoph G. Salzmann, Alexander Rosu-Finsen

**Affiliations:** a University College London, Department of Chemistry, 20 Gordon Street, London WC1H 0AJ, United Kingdom

**Keywords:** Pluto, phase transitions, methane, nitrogen, neutron diffraction

## Abstract

Maynard-Casely and co-workers [*IUCrJ* (2020). **7**, 844–851.] investigate two of Pluto’s most abundant minerals with neutron diffraction. The new results will be key to understanding the geology of our distant neighbour and represent a significant advance in the emerging field of small-molecule geology.

Far beyond the realm of the mighty gas giant Neptune lies the icy world of Pluto. Originally believed to be an inactive and desolate dwarf planet at the outer rim of our Solar system, the New Horizons spacecraft recently revealed Pluto to be a ‘scientific wonderland’ with complex surface geology (Moore *et al.*, 2016 ▸; Hall, 2020 ▸) and atmospheric chemistry (Wong *et al.*, 2017 ▸). Writing in this issue of **IUCrJ**, Maynard-Casely and co-workers have now investigated two of the most abundant Plutonian minerals, solid nitro­gen and methane, with powder neutron diffraction (Maynard-Casely *et al.*, 2020 ▸). The new results on the thermal-expansion properties, phase-transition behaviours and crystal structures of these materials will be key to understanding the active geology of our distant neighbour. This work also represents an important contribution towards advancing the emerging field of ‘small-molecule geology’ which is concerned with materials that are gases or liquids under Earth conditions but rock-forming minerals at greater distances from the Sun.

The surface of Pluto is a sight to behold. Rugged mountain ranges taller than Mount Everest give way to cracks deeper than the Grand Canyon and flat plains such as Sputnik Planitia which is part of Pluto’s famous ‘heart’ region shown in Fig. 1 ▸(*a*) (Moore *et al.*, 2016 ▸). Solid nitro­gen has been suggested to be the dominating mineral of this plain and fascinating polygonal surface patterns indicate active convection processes (Grundy *et al.*, 2016 ▸). The crystal structure of β-nitro­gen displays orientational disorder of the N_2_ molecules which are arranged in a hexagonal close-packed fashion [see Fig. 1 ▸(*b*)] (Streib *et al.*, 1962 ▸; Press *et al.*, 1982 ▸). This makes β-nitro­gen a so-called ‘plastic’ phase which is a class of mechanically soft materials and therefore consistent with the convection processes of Pluto’s plains. Maynard-Casely and co-workers present accurate density data of β-nitro­gen and follow the phase transition to α-nitro­gen which is the stable phase below 38 K (Maynard-Casely *et al.*, 2020 ▸). In α-nitro­gen, the nitro­gen molecules display defined orientations and the authors now show that its crystal structure is best described by the *Pa* 3 space group thereby resolving a long-standing debate. The phase transition from β- to α-nitro­gen takes place in a temperature range relevant for Pluto, and it can be speculated that the associated density change and most likely hardening of the material are important factors for Pluto’s glaciology.

Solid methane is expected to exist as phase I on Pluto which is, just like β-nitro­gen, a plastic phase with orientational disorder of the CH_4_ molecules that are packed in a face-centred cubic structure [see Fig. 1 ▸(*c*)] (Press, 1972 ▸). Again, Maynard-Casely and co-workers determined accurate density values for methane I which is, due to its high hydrogen content, a significantly lighter material than solid nitro­gen. To their credit, the work was carried out with protiated methane, the form of methane expected to exist on Pluto, and not the deuterated analogue (CD_4_) which would have offered tremendous advantages from the neutron diffraction point of view. Although not relevant for Pluto, they also followed the phase transition to methane II at low temperatures and observed hints of the intriguing phenomenon of negative thermal expansion. Above 65 K, they found that methane I transforms from a fine powder to large crystalline grains. Skyscraper-sized shards on Pluto are thought to consist of solid methane (Hall, 2020 ▸) and the observed changes in crystal size upon heating may provide an explanation for their formation.

Water ice is another important mineral on Pluto. Unlike solid nitro­gen and methane, its density changes very little in the temperature range relevant for Pluto (Fortes, 2018 ▸) and it is a much harder material due to hydrogen bonding between the H_2_O molecules (Salzmann, 2019 ▸). Hence, water ice is Pluto’s ‘bedrock’ material and the major constituent of its tall mountain ranges (Grundy *et al.*, 2016 ▸). The likely form of water ice to exist on Pluto is the familiar hexagonal ice I*h*. However, it has also been speculated that the ferroelectric low-temperature phase ice XI may exist (Fukazawa *et al.*, 2006 ▸).

Future work will focus on accurately measuring the mechanical properties of Pluto’s minerals. Moving beyond the traditional fitting of Bragg diffraction data, total scattering studies and the analysis of pair-distribution functions can be expected to provide detailed insights into the local structures of the orientationally disordered nitro­gen and methane phases. The interplay and mixing of the various minerals will need to be investigated in more detail as well. This will include the formation of solid solutions and the formation of clathrate hydrates in which cages of water molecules trap guest species such as nitro­gen or methane molecules. Most recently, it has been suggested that a layer of clathrate hydrates may provide the thermal insulation required to stabilize a subsurface ocean on Pluto (Kamata *et al.*, 2019 ▸). Pluto clearly still harbours many secrets and following the work of Maynard-Casely and co-workers, crystallography will certainly continue to be a major tool for understanding its geology. But also, considering how long it takes to reach Pluto, we should really send another spacecraft very soon to further explore this fascinating world.

## Figures and Tables

**Figure 1:**
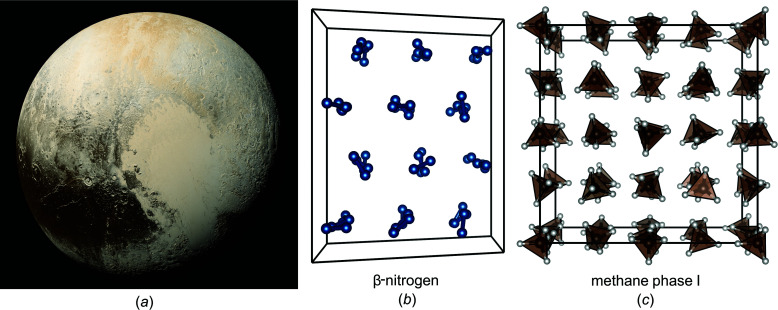
Pluto and the crystals structures of two of its most important minerals. (*a*) Photographic image of Pluto taken by the New Horizons spacecraft (Source: NASA/Johns Hopkins University Applied Physics Laboratory/Southwest Research Institute/Alex Parker. Published: July 23, 2018). (*b*, *c*) Crystal structures of β-nitro­gen(Streib *et al.*, 1962 ▸, Press *et al.*, 1982 ▸) and methane I(Press, 1972 ▸) shown with random orientations of the N_2_ and CH_4_ molecules, and using 3×3×2 and 2×2×2 supercells, respectively.

## References

[bb1] Fortes, A. D. (2018). *Acta Cryst.* B**74**, 196–216. 10.1107/S2052520618002159 29616994

[bb2] Fukazawa, H., Hoshikawa, A., Ishii, Y., Chakoumakos, B. C. & Fernandez-Baca, J. A. (2006). *ApJ*, **652**, L57–L60.

[bb3] Grundy, W. M., Binzel, R. P., Buratti, B. J., Cook, J. C., Cruikshank, D. P., Dalle Ore, C. M., Earle, A. M., Ennico, K., Howett, C. J. A., Lunsford, A. W., Olkin, C. B., Parker, A. H., Philippe, S., Protopapa, S., Quirico, E., Reuter, D. C., Schmitt, B., Singer, K. N., Verbiscer, A. J., Beyer, R. A., Buie, M. W., Cheng, A. F., Jennings, D. E., Linscott, I. R., Parker, J. W., Schenk, P. M., Spencer, J. R., Stansberry, J. A., Stern, S. A., Throop, H. B., Tsang, C. C. C., Weaver, H. A., Weigle, G. E. & Young, L. A. (2016). *Science*, pp. 351 aad9189. 10.1126/science.aad9189 26989260

[bb4] Hall, S. (2020). *Nature*, **583**, 674–678. 10.1038/d41586-020-02082-1 32728264

[bb5] Kamata, S., Nimmo, F., Sekine, Y., Kuramoto, K., Noguchi, N., Kimura, J. & Tani, A. (2019). *Nat. Geosci.* **12**, 407–410.

[bb6] Maynard-Casely, H. E., Hester, J. R. & Brand, H. E. A. (2020). *IUCrJ*, **7**, 844–851. 10.1107/S2052252520007460 PMC7467175 32939276

[bb7] Moore, J. M., McKinnon, W. B., Spencer, J. R., Howard, A. D., Schenk, P. M., Beyer, R. A., Nimmo, F., Singer, K. N., Umurhan, O. M., White, O. L., Stern, S. A., Ennico, K., Olkin, C. B., Weaver, H. A., Young, L. A., Binzel, R. P., Buie, M. W., Buratti, B. J., Cheng, A. F., Cruikshank, D. P., Grundy, W. M., Linscott, I. R., Reitsema, H. J., Reuter, D. C., Showalter, M. R., Bray, V. J., Chavez, C. L., Howett, C. J. A., Lauer, T. R., Lisse, C. M., Parker, A. H., Porter, S. B., Robbins, S. J., Runyon, K., Stryk, T., Throop, H. B., Tsang, C. C. C., Verbiscer, A. J., Zangari, A. M., Chaikin, A. L. & Wilhelms, D. E. (2016). *Science*, **351**, 1284–1293.

[bb8] Press, W. (1972). *J. Chem. Phys.* **56**, 2597–2609.

[bb9] Press, W., Janik, B. & Grimm, H. (1982). *Z. Phys. B Condens. Matter*, **49**, 9–16.

[bb10] Salzmann, C. G. (2019). *J. Chem. Phys.* **150**, 060901.

[bb11] Streib, W. E., Jordan, T. H. & Lipscomb, W. N. (1962). *J. Chem. Phys.* **37**, 2962–2965.

[bb12] Wong, M. L., Fan, S., Gao, P., Liang, M.-C., Shia, R.-L., Yung, Y. L., Kammer, J. A., Summers, M. E., Gladstone, G. R., Young, L. A., Olkin, C. B., Ennico, K., Weaver, H. A. & Stern, S. A. (2017). *Icarus*, **287**, 110–115.

